# Provision Point-Source Materials Stimulates Play in Sows but Does Not Affect Aggression at Regrouping

**DOI:** 10.3390/ani9010008

**Published:** 2018-12-22

**Authors:** Emma Catharine Greenwood, William H. E. J. van Wettere, Jessica Rayner, Paul E. Hughes, Kate J. Plush

**Affiliations:** 1 School of Animal and Veterinary Sciences, The University of Adelaide, Roseworthy 5371, South Australia, Australia; william.vanwettere@adelaide.edu.au; 2 South Australian Research and Development Institute (SARDI), Roseworthy 5371, South Australia, Australia; jessica.rayner@hotmail.com; 3 Paul Hughes Consulting, North Adelaide 5006, South Australia, Australia; paul.hughes17@bigpond.com; 4 SunPork Farms, Sheaoak Log 5371, South Australia, Australia; kate.plush@sunporkfarms.com.au

**Keywords:** aggression, group housing, mixing, point-source materials, sow

## Abstract

**Simple Summary:**

In most intensive pig production systems, pigs are often mixed into new groups. Sows are important to a production system, as they are the driver behind the farms’ productivity. The aggression that results from sows being mixed into new groups and formation of hierarchy can detrimentally affect production and welfare. This study aimed to determine the effect of providing access to materials on aggressive and play behaviors in gestating sows. Play behaviors were observed in the ‘enhanced’ pen and no play was observed in standard housing (without materials present). Aggression measures, salivary free cortisol concentrations, and injury counts were unaffected by treatment. The provision of point-source materials to sows at mixing had no impact on aggression; however, their presence maintained sow interest over the course of the experiment. Additionally, play behavior was observed in their presence, which may suggest that a more positive affect was induced when the materials were provided.

**Abstract:**

When sows are mixed into groups, hierarchies form and resulting aggression and stress can affect production and welfare. This study determined the effect of providing point-source materials on aggressive and play behaviors in gestating sows. Large white cross Landrace sows were mixed after insemination; six pens of 12 sows were housed in ‘standard’ pens, and six pens of 12 sows were housed in ‘enhanced’ pens. The ‘enhanced’ pens each contained two rubber mats, eight strands of 24 mm-thick sisal rope and two yellow plastic disks, suspended from the roof. The sows remained in these pens until pregnancy confirmation. Salivary cortisol concentration, injury counts, and sow behaviors were recorded the day before mixing (day 1), mixing (day 0) and post-mixing day 1, day 4, day 7 and day 20. At farrowing, reproductive outcomes were obtained. Play was observed (including locomotor and object play) in the ‘enhanced’ pen, and percentage of time spent playing was greater on d4 (1.48 ± 0.3 Square root transformed data (2.84% non-transformed adjusted mean)), d7 (1.43 ± 0.3 (2.97%)) and d20 (1.64 ± 0.3 (3.84%)), compared to d0 (0.56 ± 0.3 (0.70%)) and d1 (0.87 ± 0.3 (1.67%) (*p* < 0.05)). No play was observed in standard housing. Aggression, salivary free cortisol concentrations and injuries were unaffected (*p* > 0.05). The provision of materials had no impact on aggression, although their presence maintained sow interest and play behavior, suggesting a positive effect.

## 1. Introduction

In most intensive pig production systems, pigs are often mixed into new groups. Sows are important to a production system, as they are the driver behind farms’ productivity. The aggression that results from sows being mixed into new groups and formation of hierarchy can detrimentally affect production and welfare. There are many methodologies currently being tested to attempt to reduce the aggression and or resulting production effects associated with mixing. The use of enrichment materials is one possibility to assist in reducing aggression in sows. Enrichment has been defined as an addition or modification to an animal’s environment that assists its ability to cope with stressors, by promoting species-specific behavior [1,2,3]. Access to environmental enrichment has been demonstrated to reduce post-mixing aggression in finisher pigs [4,5,6,7,8]. However, the effect of enrichment on sow aggression has been less conclusive and is fairly sparse, with reports that the provision of straw bedding [9] or using spent mushroom compost on suspended wood and wire flat rack [10] reduced sow aggression at mixing; some resulted in straw having no effect on aggression [11], whereas other groups demonstrated that providing straw, either as bedding or in racks, induced competition between sows and resulted in heightened aggression [12,13]. In three recent reviews, focusing on pigs and the success of varying enrichments, the majority of studies focus on weaners or grower-finishers, with an average of 1.3% of all references focusing on sows [2,14,15]. It is difficult to draw conclusions on the large body of weaner or grower-finisher research, as the formation of sow hierarchies make it difficult to predict the effect of provided materials on aggression in sows, in comparison to younger animals. Low-ranking animals, in particular, are fearful of receiving aggression and the resulting injury, and might avoid interaction with a limited resource to reduce the risk of facing aggression [16], obscuring the value of enrichment.

The choice of enrichment for pigs in indoor systems has often been arbitrary and dependent on economic factors, rather than being designed specifically to stimulate an animal’s interest. A reported consequence of choosing enrichment on these grounds is that the animals rapidly lose interest in the objects, and this limits their value [17]. Nutritional enrichments, in the form of lick blocks, have recently been shown to decrease injury in newly mixed sows, showing that enrichment can decrease negative behaviors associated with mixing (aggression was not directly affected but only 45 min around feeding was observed) [18]; the current study chose to focus on the effects of non-nutritional, point-source materials. Some non-nutritional enrichments were more successful at maintaining attention. For example, objects suspended off the floor retained interest for longer, as devices fixed to the floor become soiled [6]. The color of the enrichment devices may also be important. Pigs have fewer cone cells than humans, and their color vision is poorer [19], so brightly colored objects might be better visualized; however, there are only a few studies that have examined pig response to color in their environments [20,21,22]. Early enrichment studies suggested that growing pigs preferred pliable or easily destructible objects [23] and orally manipulable materials [24]. Jensen’s straw enrichment (mentioned above) was widely dispersed and might have met with greater success because of this, compared to the other papers using straw, as allowed for exhibition of natural behaviors without being perceived as a limited resource [9]. However, certain housing types, such as those with slatted floors for liquid effluent systems, are obviously wholly unsuitable for this type of dispersed enrichment, and research into point-source enrichments will benefit such production systems. 

The use of man-made enrichment devices in adult sows has been scantily investigated, particularly in regards to its effects on aggression around mixing. Therefore, the aim of this study was to assess whether materials provided to newly-grouped sows could reduce aggression and in turn induce a more positive behavioral repertoire. We hypothesized that sows housed in the presence of materials (rubber mats, ropes and plastic disk swings) would engage with these materials, resulting in reduced aggressive behaviors and increased play behavior. 

## 2. Materials and Methods 

### 2.1. Animal Management

This study was conducted in accordance with the National Health and Medical Research Council/Commonwealth Scientific and Industrial Research Organization/Australian Animal Commission Australian Code of Practice for the Care and Use of Animals for Scientific Purposes [25] and with the approval of The University of Adelaide Animal Ethics Committee (Animal Ethics Number: S-2014-028B). All animal work was carried out at the University of Adelaide’s research piggery, Roseworthy, South Australia.

Animals used in the trial were from the production herd at the research piggery. In total, 144 Large White cross Landrace sows were selected at weaning and kept in stalls prior to mixing. At standing estrus, the sows were inseminated. Sows were mixed into groups of 12 sows (12 groups, 6 of each treatment) at 4 ± 1 days following the last insemination (7 ± 1 days after first detection of estrus), ensuring that there was an even parity mix across treatments (parity 1–7 and 4.3 ± 0.7). In previous parities, all sows used would have been mixed into groups after weaning and insemination. It is possible, due to the size of the facility’s herd that the sows would have previously met in previous gestation groupings, six weeks or more before our imposed mixing. Sows in this facility will have been housed in straw eco-shelters in gestation, but would not have seen other forms of enrichment prior to this trial. Sows were grouped by 0700 h on the day of mixing. The experimental period ended when all sows were scanned for pregnancy at approximately 28 days of gestation, and pregnant animals were relocated to a single straw-based shelter (at approximately 4.2 m^2^/sow) in a group of up to 40, where they remained for the remainder of gestation. If, at any point, aggression towards a certain sow was of concern, or her injuries were significantly more than the sows in the same group (evidence that a sow was being bullied), we planned to remove the sow and return her to the farm practices. However, no sows needed to be removed from a group before the experimental period ended. The study was conducted over six replicates between April and November of 2014. 

### 2.2. Housing

Pens were 8 m by 3 m (24 m^2^), allowing 2 m^2^/sow. Sows were floor-fed a standard dry sow diet (13.8% crude protein, 5% crude fiber, 0.7% lysine, 13.0 MJ DE/kg) once daily at 0700 h at a level of 2.5 kg per sow. There were two feeders in each of the pens that were cleaned and left empty for the trial (82 cm tall × 31 cm diameter; sow feeder, Rotenca, Spain). Water was available ad libitum throughout the experimental period from two bowl drinkers attached to the wall at an average of 40cm from the floor (300 mm × 200 mm bowl; ½ inch nipple bite, Mundingo, Australia). The pens were half concrete slats, and half solid concrete. The shed was naturally ventilated and during the experiment the mean daily maximum temperature was 21.3 ± 1.82 °C, the mean daily minimum temperature was 7.99 ± 1.07 °C, the mean hours of sunlight were 6.33 ± 0.34 and the mean humidity percent at 3 pm was 55.38 ± 3.30 [26]. 

### 2.3. Treatment

**Figure 1:**
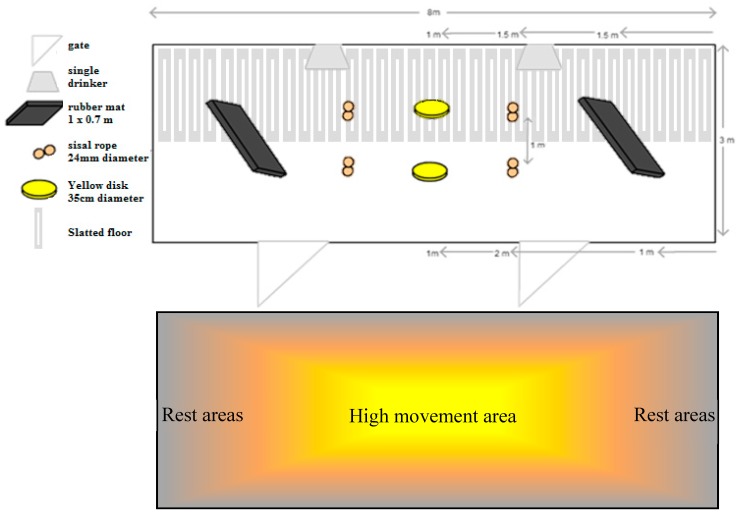
Placement of point-source materials suspended from the roof (rope, yellow disk swings and rubber mats) in the enhanced treatment pen, which housed 12 gestating sows from the point of mixing, after insemination, until pregnancy check, at a space allowance of 2 m^2^/sow. Also, a map explaining the placement of point-source materials based on predicted animal movements within the pen.

The pens were defined as either ‘standard’ (containing no added materials), or ‘enhanced’. The materials are complicated to name and so we have not called the pens ‘enriched’ as the materials provided are not strictly enrichments, but rather point-source materials used in an attempt to decrease negative interactions between sows, chasing and fleeing, frustration, and lunges and knocks and also create positive behaviors, in a pen completely designed with the intention of easing difficulties for the sows associated with mixing. The standard pens were barren pens, as described above. The enhanced pens were identical to the standard pens, except for the presence of the point-source materials. They contained two bendy rubber mats (1 cm thick, 0.7 m high × 1 m long), eight strands of 24 mm-thick, untreated sisal rope and two 35 cm diameter hard, yellow plastic disks (Childs disk swing; Swing, Slide, Climb, Sandelford Hardware Pty Ltd, Braeside, Australia). There were all suspended from the internal roof beams, with the rope knotted over the beam to create a double strand and then mats and yellow disk swings hung from chains and hooks fitted into the beams. The materials were new and clean when treatments were imposed and were not renewed over the 20-day period. The materials were hung to pig nose level, approximately 60 cm from the floor. To avoid restricting access to other limiting resources, the materials were not fitted close to walls, areas used for resting, or near drinkers. Materials that had no nutritional value were used. The materials were chosen in order to address varying negative behaviors seen in sows, evident during the process of forming hierarchies. The rubber mats were used as visual barriers, to reduce chasing behavior and allow submissive sows to escape dominant/perusing sows. The ropes were chosen, as this was likely to be one of the more successful, non-nutritional enrichments available for sows and they could be easily suspended from the roof to avoid soiling and therefore increase their longevity. This material, unlike the other two, is closer to what is expected when enriching sows (non-nutritionally). The disks were chosen in a yellow, as this is easy to see in the environment, but the disk itself was chosen in the hopes of attracting the attention of frustrated sows and perhaps defer some of the frustration and aggression, in the form of knocks mainly. The point-source materials were chosen and strategically placed in the pen in areas more suited to the behaviors that would be associated with the materials. For example, the rubber mats were placed at either end to create lying areas with visual blockages (on an angle to allow for better camera visualization). The ropes and swings were in the center of the pen and in areas where movement and interest would not cause aggression with animals attempting to rest. Details of material measurements and pen layout are shown in Figure 1, along with a map to show rationale for the placement of the individual materials.

### 2.4. Behavioral Observations

Video cameras (Legria HFR26, Cannon, Macquarie Park, Australia) were strategically fixed to allow observation of the entire pen. Sow behavior was recorded continuously for 6 h on d0, d1, d4, d7 and d20 from just prior to feeding at 0700 h until 1300 h. Sows were uniquely identified by color and symbol using stock marker (MAC tail paint and animal marker, Becker Underwood Pty Ltd, NSW, Somersby, Australia). The footage was analyzed using video analysis software (Observer XT v11.5, Noldus, Wageningen, Netherlands) using continuous sampling. Behaviors recorded included eating, drinking, active, inactive, lying, exploring floor or pen work (bars and drinkers etc.) and play and social behaviors (displacements and fighting, knocking, biting, lunging, fleeing, mounting and non-aggressive sow-sow contact). Additional behaviors relating to the provided materials were also coded. These were: play, exploration and guarding of the rope, mat or yellow disk swing (Table 1). Each behavior was either ‘mutually exclusive and continuous’ (see ethogram), or ‘point’ and so could be scored in addition to the continuous behavior. For example, during a ‘fight’ event, the sow could record a ‘knock’. The number of occurrences, event duration and the percentage of the total time spent performing each behavior was averaged across all sows within the pen for the main analysis with the pen as a unit.

### 2.5. Injury Counts 

Skin injury counts were recorded at 1400 h on day 1, day 0, day 1, day 4, day 7 and day 20 post-mixing. A modification of the assessment described by Karlen et al. (2007) was used to describe lesion counts in all treatments [2]. Each side of the sow’s body was divided into 21 areas and the number of skin lesions in each area was counted [27,28]. Sows were also placed into an injury score group and given a rank from 0–5 based on the number of injuries; sows with 0–10 injuries were group 0; 11–20 were group 1; 21–30 were group 2; 31–40 were group 3; 41–50 were group 4; 50+ were group 5.

### 2.6. Saliva Sample Collection and Analysis

Saliva samples were collected on day 1, day 0, day 1, day 4, day 7 and day 20, using cotton plugs (Salivettes, Sarstedt Australia Pty Ltd, Mawson Lakes, South Australia, Australia) attached to plastic ties. Sampling began at 1330 h on each sample day. Each sow was allowed to chew on the Salivette for a maximum of two minutes to obtain the sample [28]. Samples were centrifuged at 2012 g for 10 min at room temperature and stored at −20 °C until analysis. Saliva samples were collected on all measurement days but only samples from d-1, d1 and d7 were analyzed for cortisol levels. The samples were sent to the School of Animal Biotechnology at The University of Western Australia for analysis of salivary cortisol using MP Biomedicals I125 RIA cortisol Kit (# 07-221106) (MP Biomedicals Australia, Seven Hills, NSW). Limit of detection was 0.25 µg/dL and the mean intra and inter-assay coefficients of variation were 5.8% and 1.9%, respectively.

### 2.7. Reproductive Measures

Pregnancy rate (number of sows inseminated that were pregnant on ultrasonography at approximately day 28 of gestation), farrowing rate (number of pregnant sows that farrowed), total litter sizes, piglet weights and sex ratio of the total born litter (including stillborn, but excluding mummified, piglets) were recorded. 

### 2.8. Sow Classification Calculation

Hierarchy was assessed over two time periods: a) the number of successful displacements for each sow over all days, and b) the number of fights won and lost on the day of mixing alone. Displacements and fights were calculated as an overall or ‘global’ rank and not based on resource rank, such as displacements around food, water and space rankings, but rather all displacements in one. Hierarchy was then analyzed using two methods: first, methods from the paper by Greenwood et al. (2017) were used [29]. Briefly, the sows were separated into groups; 1D (displacement class 1) or 1F (fight class 1) sows were involved in no fights or displacements, 2D and 2F sows lost more than they won, whereas 3D and 3F sows won more than they lost. Second, the percentage fight wins for all days was calculated and sows were grouped into the following categories: ‘dominant’ won 50–100% of agonistic interactions, ‘subdominant’ won 0–49% and ‘submissive’ were involved in no fights or displacements. 

### 2.9. Statistical Analyses

Prior to analysis, data were checked for normality by examining the distribution of residual plots. This prompted us to square root transform the injury counts. Most behavioral measures and saliva cortisol concentration were log10 transformed. Where transformations occurred, non-transformed means have been presented in brackets and transformation type is specified either in tables, or parentheses. The data were analyzed using SPSS 20.0 (IBM Corp. Released 2011. IBM SPSS Statistics for Windows, Version 20.0. Armonk, NY, USA) using a linear mixed model with pen as the unit, replicate, day of measure and treatment as fixed effects and pen by day as a repeated effect. Measures tested under this model were all behaviors measured (fight number fight duration, percentage of time fighting, play and exploration, separated into explained groupings or as a total of the behavior, displacements, bite, knock, flee, lunge, active, resting, eating, mounting, sow-sow contact, guarding enrichment) total injury count, injury score, back and front injuries and salivary free cortisol concentration and reproductive data. With the exception of behavioral data, the d-1 measures were fit as covariates. Data are expressed as least squares means ± the standard error of the mean (SEM) and a *p*-value < 0.05 was deemed significant. Analysis was also conducted with sow as the unit, and is referred to as the secondary analysis, in order to analyses the effect of social rank on their response to the treatment. In these cases, sow by day was fit as a repeated effect, parity group as a fixed effect (parity 1, 2, 3 and 4+) and when hierarchy category was analyzed it was fitted as a fixed effect; this model was also used to test all variables listed in the above list. There was only one pen per treatment per replicate, thus adjusting for treatment and replicate also adjusted for the effects of pen. Cortisol results from samples of 10 and 25 µL and under were not used in the final cortisol concentration analysis, as the readings on these samples were much higher (10–400× higher) than those from samples of greater volumes (50–100 µL). For cortisol analysis, the model originally contained the time of individual sample; however, this was not significant in the model and therefore was removed. 

## 3. Results

### 3.1. Behavioural Observations 

#### 3.1.1. Fights and Displacements 

The presence of materials had no effect on the number of fights per sow per day (*p* > 0.05 enhanced; −0.40 ± 0.07 log transformed data (0.66 non-transformed mean), standard; −0.24 ± 0.08 (1.03)), on the duration of individual fights (*p* > 0.05 enhanced; 0.94 ± 0.17 log transformed data (32.6 non-transformed mean, seconds per fight), standard; 1.00 ± 0.16 (37.76)) or on the percentage of total time spent fighting (*p* > 0.05 enhanced; −1.56 ± 0.14 log transformed data (0.14 non-transformed mean, proportion), standard; −1.46 ± 0.14 (0.09)). Fight number was affected by the number of days following mixing, with more fights recorded on d0 than any other day (*p* < 0.001). Similarly fight duration was longer on d0 than any other day (*p* < 0.001). The overall percentage of time spent fighting was greater on d0 compared to other days, and greater on d1 compared to d20 (*p* < 0.001, Table 2). 

The number of displacements averaged per sow per day was not affected by the presence of materials (*p* > 0.05, enhanced; 01.26 ± 0.84 SQRT (square root) transformed data (1.88 non-transformed mean), standard; 1.44 ± 0.84 (2.42)). The number of days after mixing affected displacements, with more displacements occurring on d0 than all other days and more displacements occurring on d1 and d4 compared to d7 and d20 (*p* < 0.001, Table 2). 

#### 3.1.2. Bites and Knocks 

Bite number was unaffected by treatment (*p* > 0.05 enhanced; 0.59 ± 0.05 log transformed data (7.90 non-transformed mean), standard; 0.66 ± 0.05 (7.92)) but was impacted by day after mixing, with higher bite number on day 0 than all other days and lower bite number on day 20 then on d1 and 4 (*p* < 0.001, Table 2). The overall number of knocks delivered was not altered by treatment (*p* > 0.05) but was by day, with increased knock number on d0 than all other days and lower knock number on day 20 compared to day 4 and day 7 (*p* < 0.001, Table 2). The interaction of treatment by day was not significant for knock number (*p* < 0.05). No aggression as a direct result of the objects was observed. 

#### 3.1.3. Eating and Inactivity 

The percentage time spent eating was not affected by treatment (*p* > 0.05) or day (*p* > 0.05). The percent time spent inactive was affected by day (*p* < 0.001), with the lowest percentage of time spent inactive being on d0 (45.1 ± 2.5%) compared to all other days, and day 20 (56.5 ± 2.3%) compared to day 1 (64.3 ± 2.3%), d4 (61.5 ± 2.6%) and day 7 (65.5 ± 2.6%). 

#### 3.1.4. Play and Exploration 

**Figure 2:**
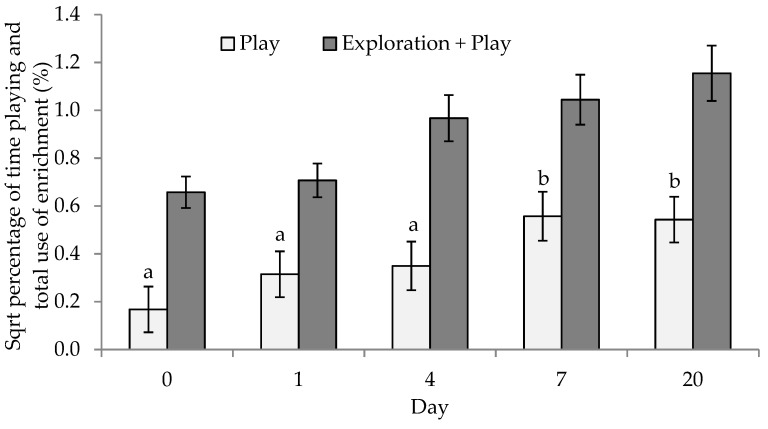
The effect of housing on mean ± SEM (standard error of the mean) percent of total time in play and the total time using the materials (play + exploration; over 6 h, from 0700 hours) in enhanced housing (hanging mats, ropes and yellow disks), of group housed sows following mixing (d0) in (significant differences are indicated by different superscripts, play; ^a, b^ *p* < 0.001, total material use (play + exploration) *p* > 0.05).

The standard pen recorded no play events across all days examined; with the enhanced group displaying a greater percentage of time spent playing on all days (*p* < 0.001). The percentage of time spent playing was increased within the materials treatment across days, with significantly higher percentage of time spent playing on day 4, day 7 and day 20, compared to day 0 and day 1 (d0: 0.56 ± 0.3 (0.70%), d1: 0.87 ± 0.3 (1.67%), d4: 1.48 ± 0.3 (2.84%), d7: 1.43 ± 0.3 (2.97%) and d20: 1.64 ± 0.3 (3.84%), *p* < 0.001, Figure 2).

The percentage of time spent exploring alone was not affected by treatment (*p* > 0.05 sum of materials, floor and penwork). The total time spent interacting with the materials (play + exploration) pooled across days was 1.7% (non-transformed mean, Figure 2). This equated to an average of 6.12 min of material use per sow per pen during the 6 h recording period. The total time spent with the provided materials (sum of play and exploration) was not significantly altered by day (*p* > 0.05). Overall, the rope was the most used material (Table 3).

#### 3.1.5. Time Budgets

Analysis of all behaviors separately are discussed above. Below is Table 4, which lays out each continuous behavior, analyzed, showing overall percentages over the 6-h observation period spent in each behavior, separated by day and by treatment. 

### 3.2. Injuries

The number of injuries sustained by sows in groups was not altered by treatment within day (*p* > 0.05), or treatment alone (*p* > 0.05 enhanced; 4.98 ± 0.17 SQRT transformed data (25.83 non-transformed mean), standard; 5.25 ± 0.17 (28.75)). When injury scores were grouped into severity (0–5), there was also no difference across treatments (*p* > 0.05 enhanced; 2.06 ± 0.13, standard; 2.13 ± 0.13). The number of injuries was shown to differ across day of measurement (*p* < 0.005), with lower injuries on day 0 than day 1, day 4 and day 7 and lower injuries on day 20 compared to all other days (Table 2). 

### 3.3. Salivary Cortisol Concentration

Salivary cortisol concentration was not affected by treatment (*p* > 0.05, enhanced 1.32 ± 0.04, 34.0 ng/mL and standard 1.37 ± 0.04, 26.1 ng/mL), or day (*p* > 0.05, d1 = 1.3 ± 0.06 (32.3 ng/mL), d7 = 1.4 ± 0.06 (27.9 ng/mL)). 

### 3.4. Sow Weight and Reproduction 

There was no effect of treatment on the change in weight from day 1 to day 20 after mixing (*p* > 0.05). Due to problems with on-farm fertility, pregnancy rate was low (72.4 ± 9.1%), but was unaffected by treatment (enhanced = 68.0 ± 3.6%, standard = 77.8 ± 3.6%, *p* > 0.05). The total number of piglets born per litter (enhanced = 12.2 ± 0.7, standard = 13.4 ± 0.7), average piglet weight (enhanced = 1.4 ± 0.05, standard = 1.4 ± 0.05) and sex ratio of the litters (percentage of the litter female, enhanced = 44.7 ± 2.7, standard = 51.0 ± 2.7) were also not affected by treatment (*p* > 0.05). 

### 3.5. Hierarchy 

Neither hierarchy analyses (DF ranking (our ranking based on displacements and fights) or dominant, subdominant, submissive) revealed an effect of hierarchy on the use of materials, either the percentage of time spent playing with (*p* > 0.05), or the percentage of time exploring the materials (*p* > 0.05). 

## 4. Discussion

The presented data partially support our hypotheses that sows housed in the presence of materials (rubber mats, ropes and plastic disk swings) would engage with these materials, resulting in reduced aggressive behaviors and increased play behavior. Although there was no impact of the materials employed in this experiment on aggression and stress in grouped sows, we did observe that the added materials resulted in exploration and play. Play in particular is important to show that the materials are of use, as the animals are likely to explore any new material in the pen, but the presence of play highlights its merits. These behaviors indicate that a more positive affective state was induced in the enhanced pen sows; however, further work to confirm this statement is required. We did not decrease aggression with the provision of materials aimed at doing so. However, play was created by the presence of the materials, showing positive effects of the presence of the materials.

### 4.1. Effect of Pont-Source Materials on Aggression and Injuries

The provision of point-source materials exerted no overall effect on aggressive behavior in the newly mixed sows. It would be easy to conclude that the materials used did not interest the sows, but the data show that sows interacted with the items, so the lack of any treatment effect on fighting behaviors might be explained by the level of motivation for different limiting resources. In a recent study by Horback et al. (2016), where similar materials were employed (hanging ropes, hanging rubber sticks and a fixed wooden block), similar results were obtained; the materials were used, but no significant differences in lesion severity or sow activity resulted [30]. This study placed each different material in separate pens and allocated no barren pen, thus compared the success of the material separately. The role of our rubber mat in particular would have been interesting if we could have further assessed its success alone in decreasing fleeing behavior through creating visual barriers. In studies in younger pigs visual barriers have been found to decrease negative effects of group housing and mixing, with decreased injury and belly nosing (signifying a decrease in stress) [31] and with aggressive interactions decreasing 40% [32]. Interestingly, the same study that found 40% reduction with visual barriers found no difference between aggression in straw based or barren housing [32]. However, this was not successfully analyzed in the current trial as the behaviors coded did not allow us to fully separate the effect of the mats alone. It would have been interesting if like the previously mentioned study we also had individual pens with each enrichment as well as our current treatments. Horback et al. (2016) concluded that the drive to achieve social hierarchy took precedence over interest in the materials [30]. This might explain why, in our study, material use increases with time after mixing, since hierarchies have formed and started to stabilize, the sows can be more easily distracted. This suggests that it might not be possible to decrease mixing aggression in sows by providing material distractions, but that they may have a role later in generating a ‘positive affective state’. 

In a study using operant conditioning, Pedersen et al. (2002) investigated how hard a pig would work for certain resources (by pressing a button with their snout), both in isolation, and with the presence of a companion pig [33]. The experiment demonstrated that in the presence of a companion pig, the motivation for food increased, but the motivation for straw did not. The sow’s motivation for food likely outweighs any other environmental factors when housed in groups. Interestingly, when in solitary conditions, Hemsworth et al. (2011), who found that in a Y maze test, sows worked harder to be back with another pig than for food when in solitary conditions, showing the importance of social interaction to the animals [34]. Whilst the behavioral recordings in the present study were collected over a 6-h period, which commenced at feeding, aggression at feeding versus a later time in the presence and absence of the materials should be explored. Alternatively, the materials used in the present study could be tested for effectiveness under a less-competitive feeding regime (such as ad libitum access, or where electronic sow feeders are used). 

The materials did not increase aggression in this study, in contrast to a previously published study demonstrating that sow-sow aggression increased when enrichment was provided in the form of straw in racks [13]. This was attributed to the straw being considered a limited resource by the sows. This was carefully avoided in the present investigation by providing the same number of pieces of material as there were sows in the group, and ensuring that the materials were not situated in areas where sows would rest, or by drinkers; thereby avoiding obstruction to other limiting resources. Elmore et al. (2011) also examined point aggression in sows, comparing usage across social rank rather than to a non-enriched control. Subordinate sows used enrichment more in non-peak times, suggesting another area of concern with using enrichment in sows [3]. However, both hierarchy analyses conducted in this experiment found that there was no difference in material usage across hierarchy groups. The usage was the same in subordinate sows in the present investigation, perhaps due to the spread and number of materials. 

In general, enrichment has been associated with an increase in cortisol [35,36,37], but this increase in hypo-pituitary-adrenal (HPA) axis activity was not observed in this study. Higher cortisol levels observed in previous studies may reflect the increased movement and activity, as movement increases metabolic demand, which, in turn increases cortisol secretion. Cortisol is a primary metabolic hormone, with its main role being the metabolism of energy [36]. The seemingly higher cortisol in enriched pigs may also be due to a blunted cortisol circadian rhythm in standard housed animals [35]. This phenomenon may be related to excitement or enjoyment of the materials provided, as cortisol rises in response to both positive and negative situations [38]. This response was not seen in our sows, possibly because a single saliva sample for free salivary cortisol concentrations per day was insufficient to detect changes in HPA axis activity between the two treatments. However, this cannot be entirely accepted, as Yang et al. (2018) found an effect at weaning in piglets’ cortisol in enriched vs non-enriched, with just a pre-weaning and post-weaning sample [39]. 

Hormones associated with stress can impair reproduction in sows [40,41,42]. Given that no differences in aggression, injury or cortisol concentration were observed between the standard and enhanced treatment pens, there was no effect on any reproductive trait measured. These results concur with others, which show reproduction of pigs in alternative enriched housing systems to be similar, and in some cases better, when compared with barren systems [2]. Sex ratio of the litter was recorded out of interest, as it has long been suggested that stress in gestation may result in sex-specific losses, due to males’ generally higher developmental rates and therefore different responses to stressors in utero [43]. In this measure too, there was little impact of material usage, which was not surprising given the failure of the treatments to impact on aggression.

### 4.2. Days after Mixing 

Irrespective of the effect of material provision on sow behavior, it is clear that aggression was greatest on the day of mixing, and decreased considerably thereafter. Other studies have demonstrated a reduction in aggression as early as one hour following mixing [44], but it is generally agreed that in domesticated sows, dominance hierarchies form within 2 to 10 days of mixing [45,46,47,48]. It would appear that in the present study, the social rank (and thus level of aggression) was stabilized after the first day of the mixing event. Additionally, play behavior increased following mixing and the proportion of time spent playing almost tripled by day 7 after mixing and was still more than double by day 20. This is an important finding, as it is well known that aggression will peak at regrouping, but less is known about more positive social interactions and when and how they are exhibited following mixing. Our time budget table also highlights subtle differences between the behaviors in the two groups over all days. 

### 4.3. Success of the Materials in Creating Posative Behaviours

Enrichment is generally provided on a species-specific basis, based on what research suggests that particular species requires and on our knowledge of their behavior in the wild, and in captivity. However, assessment of the success of any given enrichment has been minimally discussed in the literature in regards to sows in particular. Generally, the worth of a material is measured by observing the ethogram of captive animals in comparison to their wild counterparts [49], by preference testing [24,25] or assessing cognitive bias [50]. The presence of the materials did not affect aggression but maintained sow interest, with interaction with the enrichment increasing over 20 days and creating play behavior. We were not expecting that these materials would maintain the interest of the sows for even 7 days, but interest and use maintained up to 20 days was very unexpected. Notably, play behavior was not observed in the barren housing environment. There was a chance that aggression would be increased by the presence of our materials (resource competition), even though we did not see this with our enrichments. For this reason, point-source enrichment should not be recommended to producers currently for addressing sow aggression specifically. Research into our materials did aid in understanding the properties of materials that interest sows, into which there is minimal research, and that may be of value in Australian systems with slatted effluent management in the future. 

There is increased interest in welfare research into behaviors that are assessed as positive, rather than just a decrease in negative behavior. Positive behaviors include play, foraging, exploration and affiliative interactions [51]. In a review, Boissy et al. (2007, pg. 375) states that ‘good welfare is not simply the absence of negative experiences, but rather is primarily the presence of positive experiences, such as pleasure’ [52]. Play is one of a few behaviors often cited as a marker for positive experiences in farm animals under commercial conditions [51]. Where play in young animals is obvious and often has an experiential and educational role, play in adult animals is less commonly observed and can be considered to be behavior without survival need. The present enrichment created play behavior in sows, which could possibly suggest a positive welfare state. Held and Špinka (2011) reviewed the complexities of the play-welfare relationship [53]. They stated that play indicated the absence of fitness threats, acts as a reward and induces opioid-mediated pleasurable emotional experience, brings immediate psychological benefits and long-term fitness and health benefits, ‘is socially contagious and therefore capable of spreading good welfare in groups’ ([52], pg. 891). Play in piglets and other juvenile animals is widely considered as a positive welfare marker, which can enhance physical development and create social bonds [54]. It has been suggested that there are several ways that play can be linked to welfare state, play can lead to or cause increased welfare, reduced playfulness can indicate reduced welfare and play can reflect the presence of positive or good welfare [55]. 

However, this is still a complicated relationship, as play can also be present in stressful situations, for example after weaning in piglets in response to removal from maternal care [52]. In adult animals, play is less frequent and any apparent benefit would be short term [56]. It would seem that adult play is present commonly in conjunction with stress and arousal [56]. It may be possible that adult play is a sign of altered welfare which animals try to compensate for when given the opportunity [56]. This view makes our results more complicated, as it is more difficult to decide if our increase in play is linked to an increase in welfare state. As all sows in this experiment experienced the same stressors, and we know that a stressor has been imposed in the form of mixing. Those on the experimental treatment group alone exhibited play behaviour and therefore, it seems reasonable to conclude that the play behavior was either a positive welfare response to the presence of the materials or a coping mechanism to stress, which the sows have the opportunity to utilise due to the presence of the provided point-source materials. 

Play behavior in the sows provided with materials increased over the 20-day experimental period, indicating that the materials were an increasing source of interest to the sows. This increase in play in the days further from the mixing may highlight a good welfare state more than analyzing the play around mixing alone, as the play behavior was still exhibited and also increased further away from the imposed stressor. The choice of enrichment for pigs in indoor systems is often arbitrary and dependent on economic factors, rather than the requirements of the animals themselves. A consequence of choosing enrichment on these grounds is that the animals commonly rapidly lose interest in the objects, and this limits their value [17]. Our enrichment was chosen after studying previous research (in grower-finishers predominantly) with an attempt to increase longevity of the material usage. All of the enrichments were hanging, with no contact with the floor, as in previous trials hanging or suspended objects retained the animals’ interest for longer [as when fitted to the floor the enrichment became soiled, [6]. Growing pigs prefer pliable or easily destructible toys [23] and orally manipulable materials such as ropes rather than rubber strips [24]. For these reasons, we anticipated that our materials would maintain the sows’ interests longer than other material options. However, it is difficult to say that the preferences of grower-finisher pigs (on which the majority of enrichment research is based) can be passed onto sows and the assumption that the same materials be biologically relevant to the different ages may not be correct. However, this research has resulted in positive play behaviors and sustained use (although not reduced aggression which was our main aim with the materials given). Further research is definitely needed into the materials which will be optimum to enrich sows and of huge importance is that these provided materials can be shown to increase sow welfare and at different stages in their cycle. 

## 5. Conclusions

Currently, it is difficult to argue for the use of point-source materials in intensive farming methods, even though these methods are easier to manage than bedding, for example, as enrichment. At what point the cost of the enrichment is outweighed by its value to the animal is still difficult to determine. Sow aggression was unaffected by the provision of materials the days after a mixing event. Play behavior was created in the sows provided with point-source materials, and this could indicate a more positive welfare state or a coping behavior made possible by the presence of the materials. The fact that the materials provided did not increase aggression between the sows was a positive finding, as although interest in the materials is evident it may not have been perceived as a limiting resource. In conclusion, the creation of play behavior may suggest that the welfare of sows was improved when materials were present during the mixing event.

## Figures and Tables

**Table 1:** Ethogram for analysis of sow behavior.

Active, lying, inactive, eating and drinking	Sows were classed as eating if food was present and she was noted as collecting food from the floor, chewing and/or swallowing. The sow was drinking if her head was in the drinker and she could be seen to swallow and/or actively manipulate the drinker nipple. If it was unclear what the sow was doing and if she was dog-sitting, standing or walking she was considered active. Sows were coded as lying down if lying flat to the floor either on their side or on their belly. Sows were considered inactive if lying down.	Continuous
Exploring	Actively manipulating and exploring the surrounding environment, such as rooting, nosing the floor, moving drinkers and chewing fences. When concerning the materials, this was coded when the animal manipulated the rope, disk swing or mat (mainly chewing or nosing the material). Exploration is analysed for separate material use, overall material use, and exploration, except for materials and exploration total.	Continuous
Fighting	Aggression including three or more knocks or bites. Aggression can be reciprocal or non-reciprocal and was coded from a sow adopting a parallel pushing defensive posture as well as bite or knock interactions.	Continuous
Play	All locomotor and object play were included in one overall heading of ‘play’. Locomotor play included running, hopping (focal animal has either its two front feet or all four feet off the pen floor at one time through an energetic upwards jumping movement.), pivots (twirling of body on the horizontal plane by a minimum of 90° usually associated with jumping on the spot) and head tossing (quick and often erratic shaking of the head back and forth and up and down). Object play is when an animal manipulates an item or securely holds it in its mouth, energetically shaking it [1]. Object play was specifically coded if directed towards a specific object to allow analysis of play around/using the enrichment (rope, yellow disk swing, mat and play not centred on a specific object or other).	Continuous
Displacement	Movement of one sow by another, from a valued resource such as food, drinker or lying space (if multiple knocks or bites are required, this is a fight)	Point
Knock	One sow knocks another sow using her head and neck, contacting any part of the receiving sow	Point
Bite	One single bite delivered from one sow to any part of another	Point
Lunge	Sow lunges at another but does not make physical contact	Point
Flee	Sow moves herself quickly and as far away as the can get from another sow, in response to an aggressive action	Point
Mounting/mounted	One sow mounts another, with her front legs both over the back of the other animal. This behaviour was coded as long as the mounting animal remained on top of the mounted. Mounting and mounted were both scored as separate behaviours.	Point
Non-aggressive sow-sow contact	Mutual contact between two sows, which involves exploration of another animal with no aggressive outcomes (does not include lying with another sow)	Point
Guarding enrichment	Any form of aggression (knock, bite, lunge) or defensive posturing to stop other sows from the use of enrichment, whether resulting in a fight or displacement from enrichment. This behaviour could fall under displacement or fight if it fit into the below description, but was also specifically allowed for separately, in order to allow analysis of any aggression surrounding the enrichment (sows could be coded as guarding individual enrichment items, mat, disk swing or rope).	Point

**Table 2:** The effect of day after the mixing event on parameters linked to aggression, including different behaviors and injury counts per sow in sows housed in groups of 12 from the day of mixing to day 20 following mixing *^,1^.

Day	0	1	4	7	20	Tran.	*p*-Value
Fight number	0.4 ± 0.1 ^a^ (3.4)	−0.4 ± 0.1 ^b^ (0.3)	−0.5 ± 0.1 ^b^ (0.3)	−0.7 ± 0.1 ^b^ (0.2)	−0.5 ± 0.2 ^b^ (0.1)	Log^10^	<0.001
Fight duration, sec	1.9 ± 0.2 ^a^ (127.6)	0.9 ± 0.2 ^b^ (13.0)	0.8 ± 0.2 ^b^ (10.8)	0.7 ± 0.3 ^b^ (14.9)	0.6 ± 0.3 ^b^ (9.7)	Log^10^	<0.001
Time spent fighting, %	−0.4 ± 0.2 ^a^ (0.5)	−1.4 ± 0.2 ^b^ (0.06)	−1.8 ± 0.2 ^b^ (0.03)	−2.1 ± 0.3 ^b^ (0.1)	−1.9 ± 0.3 ^b^ (0.1)	Log^10^	<0.001
Displacement number	2.0 ± 0.1 ^a^ (4.2)	1.3 ± 0.1 ^b,c^ (1.9)	1.4 ± 0.1 ^b^ (2.2)	± 0.1 ^c^ (1.3)	1.1 ± 0.1 ^c^ (1.1)	SQRT	<0.001
Bite number	1.3 ± 0.1 ^a^ (25.1)	0.6 ± 0.1 ^b^ (4.9)	0.6 ± 0.1 ^b^ (4.5)	0.4 ± 0.1 ^c^ (2.9)	0.3 ± 0.1 ^c^ (2.3)	Log^10^	<0.001
Knock number	1.1 ± 0.1 ^a^ (14.5)	0.6 ± 0.1 ^bc^ (4.0)	0.7 ± 0.1 ^b^ (5.4)	0.7 ± 0.1 ^b^ (4.6)	0.5 ± 0.1 ^c^ (3.4)	Log^10^	<0.001
Injury count	4.9 ± 0.2 ^a^ (24.7)	5.5 ± 0.2 ^b^ (31.6)	5.5 ± 0.2 ^b^ (31.3)	5.4 ± 0.3 ^b^ (30.9)	4.1 ± 0.2 ^c^ (17.9)	SQRT	<0.05

Significant differences within row are indicated by superscripts (^a, b, c^ *p* < 0.05). * Mean and SEM (standard error of the mean) presented; with non-transformed, adjusted means presented in parenthesis; ^1^ Behaviors were recorded for 6 h daily from 0700 h; Tran.: Transformation used; SQRT: square root.

**Table 3:** The SQRT transformed proportion of use, for each material provided (rope, mat and yellow disk swing) and the amount of play and exploration calculated per sow *^,1^.

Behavior	Total Time in Play, %	Total Time Exploring, %
Rope	1.4 ± 0.1 (2.4)	0.9 ± 0.2 (1.1)
Mat	0.2 ± 0.1 (0.3)	0.5 ± 0.3 (0.5)
Yellow disk swing	0.3 ± 0.1 (0.1)	0.1 ± 0.1 (0.1)

* Mean and SEM presented; with non-transformed, adjusted means presented in parenthesis; ^1^ Behaviors were recorded for 6h hours daily from 0700 hours.

**Table 4:** The proportion of time spent in continuous behaviors in sows in enhanced and standard group pens in the days following a mixing event *^,1^.

Behavior	Treatment	Day 0	Day 1	Day 4	Day 7	Day 20
Active	enhanced	35.55 ± 2.23	20.21 ± 2.11	22.64 ± 2.33	18.69 ± 2.32	27.22 ± 2.11
	standard	33.66 ± 2.16	18.31 ± 2.11	20.74 ± 2.33	16.79 ± 2.32	25.32 ± 2.11
Resting	enhanced	43.79 ± 2.68	62.89 ± 2.54	60.06 ± 2.81	64.12 ± 2.79	55.13 ± 2.54
	standard	46.59 ± 2.60	65.69 ± 2.54	62.87 ± 2.80	66.93 ± 2.79	57.93 ± 2.54
Eating	enhanced	5.43 ± 0.52	6.55 ± 0.49	7.09 ± 0.54	6.49 ± 0.54	6.98 ± 0.49
	standard	5.08 ± 0.51	6.21 ± 0.49	6.75 ± 0.54	6.15 ± 0.54	6.64 ± 0.49
Exploring	enhanced	3.16 ± 0.13 (10.28)	2.75 ± 0.12 (7.72)	2.65 ± 0.14 (7.3)	2.65 ± 0.14 (7.35)	2.96 ± 0.12 (8.93)
	standard	3.0 ± 0.13 (9.46)	2.59 ± 0.12 (6.9)	2.49 ± 0.14 (6.48)	2.52 ± 0.14 (6.53)	2.08 ± 0.12 (8.12)
Fighting	enhanced	0.51 ± 0.07	0.08 ± 0.06	0.05 ± 0.07	0.01 ± 0.07	0.03 ± 0.06
	standard	0.46 ± 0.07	0.04 ± 0.06	0.00 ± 0.07	0.01 ± 0.07	0.01 ± 0.06
Mounting	enhanced	1.42 ± 0.34	0.09 ± 0.33	0.16 ± 0.35	0.53 ± 0.35	0.09 ± 0.33
	standard	1.41 ± 0.33	0.08 ± 0.33	0.16 ± 0.35	0.52 ± 0,35	0.08 ± 0.33
Play	enhanced	0.3 ± 0.11 (1.46)	0.6 ± 0.12 (1.65)	1.1 ± 0.12 (2.55)	0.7 ± 0.12 (2.22)	1.1 ± 0.12 (2.98)

* Mean and SEM presented; with non-transformed, adjusted means presented in parenthesis, play and exploration data are SQRT transformed; ^1^ Behaviors were recorded for 6 h daily from 0700 hours.
